# An Experientially Derived Model of Flexible and Intentional Actions for Weight Loss Maintenance After Severe Obesity

**DOI:** 10.3389/fpsyg.2019.02503

**Published:** 2019-11-13

**Authors:** Eli Natvik, Målfrid Råheim, John Roger Andersen, Christian Moltu

**Affiliations:** ^1^Department of Health and Caring Sciences, Western Norway University of Applied Sciences, Førde, Norway; ^2^The Centre for Health Research, District General Hospital of Førde, Førde, Norway; ^3^Department of Global Health and Primary Care, University of Bergen, Bergen, Norway; ^4^Division of Psychiatry, District General Hospital of Førde, Førde, Norway

**Keywords:** weight loss, weight loss maintenance, long term, behaviour change, self-management, experience, qualitative research, self-determination theory

## Abstract

**Background:**

Knowledge about *non-surgical* weight loss (WL) is scarce among people with severe obesity (SO). Lifestyle changes are primarily self-driven, occasionally accompanied by professional guidance and weight-management support. Weight regain and intervention discontinuation are common challenges among guidance and support programmes. In the current study, we describe a model of meaningful strategies for maintaining WL after SO based on the experiences of successful cases.

**Methods:**

Aiming to investigate the experiences of WL and weight loss maintenance (WLM) (≥5 years) following SO, we designed a qualitative study. Ten adults of Norwegian ethnicity, eight women and two men aged from 27 to 59, participated in individual in-depth interviews. We recruited participants living in rural districts and cities across all four regions of Norway. The interviews concentrated on participants’ experiences of losing weight and maintaining a lower weight over the long term. The transcripts were analysed with a rigorous method for thematic cross-case analysis, namely, systematic text condensation (STC).

**Results:**

Participants identified four experiential themes at the core of long-term WLM: (a) *Owning the decision*, (b) *Creating self-reinforcement*, (c) *Sustaining a lifestyle-forming identity*, and (d) *Selecting support appropriate to one’s own situation.* These core themes represent *the intentional level*, functioning both as the foundation of and the momentum for sustaining WL. On the *behavioural* level, participants continued to take action for change, obtain results, record and reflect on their efforts and milestones, observe what worked and felt good, and receive recognition from others, thereby realising changes.

**Conclusion:**

Based on these results, we propose a model of WLM after SO, suggesting that practices toward WLM on the behavioural level achieve meaning and sustainability through their relationship with a core intentional level found across participants’ experiences. One implication is that the relationship between the intentional and behavioural levels might be more meaningful when discussing long-term WLM than the behaviours themselves.

## Introduction

Achieving long-term weight loss (WL) through changes in eating and physical activity (PA) is possible for individuals with severe obesity (SO; BMI ≥35–40) (Ryan et al., 2010). SO reduces life expectancy and significantly threatens vital life functions (Kitahara et al., 2014; World Health Organization, 2017); a WL of 10–20% of one’s initial body weight for ≥1 year is vital for improved health (Wing and Phelan, 2005; Yumuk et al., 2015). Weight loss maintenance (WLM) is critical to maintain these health benefits; however, only 20% of those who lose weight successfully keep the weight off for ≥1 year (Wing and Phelan, 2005; Dombrowski et al., 2014; Thomas et al., 2014). Most people regain lost weight, and many resume old diet and PA habits during an early phase (Byrne et al., 2003; Ryan et al., 2010; Look Ahead Research Group, 2014; Thomas et al., 2014).

Bariatric surgery is an appropriate and effective intervention for WL among people with SO. However, this procedure is not available worldwide, has potential risks and side-effects, and is not preferred by all people who present with SO. Consequently, individuals with SO attempting to lose weight need appropriate and effective non-surgical alternatives as well as self-guided approaches to cope with excess weight (Middleton et al., 2012; Teixeira et al., 2012; Gorin et al., 2014). People who have lost weight successfully must create permanent healthy diet and PA routines as well as overcome the physiological, psychological and environmental factors that lead to weight regain (Stubbs et al., 2011). WLM is a challenging, potentially stressful lifelong task that takes time and effort to establish (Greaves et al., 2017; Natvik et al., 2018). Research indicates that professional support for WLM is effective, but these programmes are not widely distributed, and results are inconsistent with regard to their design and delivery (Wing et al., 2006; Middleton et al., 2012; Gorin et al., 2014; Lean and Hankey, 2018). Therefore, understanding how best to support people maintaining WL and the long-term health benefits are crucial for health practitioners helping patients with obesity.

One systematic review of randomised controlled trials showed small but significant effects regarding WLM following *non-surgical* interventions that targeted both diet and PA for 2 years (Dombrowski et al., 2014). An older meta-analysis indicated that 4 or 5 years after participating in a structured WL programme, the average WLM was 3 kg (3.3% of initial body weight), and 7 kilos (6.6%) among individuals who had participated in very low calorie diets (VLCDs) or lost ≥20 kg (Anderson et al., 2001). However, the findings regarding VLCD are inconsistent. One clinical study found that individuals using a self-directed approach maintained their initial WL with greater success than those who had used a VLCD (Pinto et al., 2008). Furthermore, clinical and registry studies tend to report greater long-term success. The Look AHEAD study reported ≥5% WL for 50% of overweight/obese adults with type II diabetes 8 years after a comprehensive lifestyle intervention (Look Ahead Research Group, 2014). Based on the National Weight Control Registry (NWCR), a prospective study reported that the majority of weight lost via lifestyle change was maintained 10 years later, with an average WL of 22.6% from maximum weight at 5 years but no significant change at 10 years (Thomas et al., 2014). These findings support the conclusion that interventions for WL and WLM are beneficial.

Many factors associated with successful WLM have been reported such as reaching a self-determined weight goal early; regular PA; eating regular meals; reducing energy intake; increasing dietary fibre, fruit, and vegetable consumption; self-monitoring; scheduling; internal motivation; coping strategies and ability to handle life stress; self-efficacy; autonomy, psychological strength and stability; professional support; and management aids (Elfhag and Rössner, 2005; Stubbs et al., 2011; Coughlin et al., 2013; Ramage et al., 2014; Hartmann-Boyce et al., 2017). Many factors are correlated with success; however, the variance that they explain is either small or highly variable between groups.

As mentioned, most attempts at WL and WLM fail. In a systematic review of high-quality studies, Santos et al. (2017) found that approximately four in 10 adults have tried to lose weight at some point over the last 5 years. The contradiction between the prevalence of WL attempts and of successful long-term WLM indicates that people aiming for WL and WLM mostly do so without taking part in interventions and programmes. Self-directed approaches can produce modest WL in the short term, although adding other interventions is beneficial to achieve clinically meaningful WLM (Tang et al., 2016). One systematic review of the qualitative research on self-directed WL and WLM investigated self-monitoring (Hartmann-Boyce et al., 2019). The results indicated that self-monitoring is related to self-perception and emotions and can be helpful. However, it also risks inducing shame and the urge to quit. A recent systematic review of qualitative studies examined the concept of *reframing* with regard to self-directed WL and WLM (Hartmann-Boyce et al., 2018). Reframing refers to changing one’s way of thinking about WL and WLM to make it more achievable, durable, and less challenging; this concept is similar to cognitive restructuring. The results indicated that people who intentionally switch from perceiving WL and WLM as dieting to conceiving it as a new way of living, expressed fewer negative emotions and maintained their efforts.

Knowledge about successful WL and WLM among people with SO might be useful for health practitioners supporting people with weight-related issues. Trying to accommodate radical life changes beyond dietary restrictions and exercise routines is a challenge and calls for understanding, knowledge and long-term support. This goal is particularly important because most people aiming for WL and WLM use self-directed approaches rather than programmes and interventions as well as seek support from practitioners to whom they have access. Research describes a range of factors associated with successful WL and WLM; however, why some attempts are successful but most fail is not fully understood. Understanding the experiential processes involved in successful WLM might aid the development of professional support for obesity. This qualitative study investigated the experiences of a group of successful WL maintainers who previously had SO, lost weight successfully, and maintained a lower weight for at least 5 years. Research from the first person perspective is particularly important in this context, because the experience of people who maintain clinically significant (non-surgical) WL seldom gain significance beyond popular media. Although success stories are familiar in obesity practice and research, they are often disregarded as just anecdotal (Freedhoff and Hall, 2016). Hence, an in-depth exploration to provide a rich description with concrete examples can change current discussions about WLM, interventions for WL and support for people engaging in self-directed WL.

Aiming for a nuanced and insightful understanding of long-term WLM, the current study provides experiential descriptions and proposes a conceptual model for further research. We explore the following research questions: How do weight loss maintainers who previously had SO experience changes and continuity 5 years after initiating successful WL? What are the experiential drivers of successful WL and WLM after SO?

## Materials and Methods

### Participants

Ten participants with previous SO, ranging in age from 27 to 59 and with a current self-reported BMI ranging from 25.8 to 39.1 kg/m^2^, participated in individual in-depth interviews. The participants lived in rural districts and cities across six counties and all four regions of Norway. Table 1 presents the demographics and clinical characteristics of the participants.

**TABLE 1:** Demographic and clinical characteristics of participants.

**Characteristics**	**Count**
**Gender**	
Male	2
Female	8
**Age**	
25–30	3
30–35	1
35–40	3
40–45	2
55–60	1
**Cohabiting status**	
Married/cohabiting with children	6
Married/cohabiting	2
Living alone	2
**Education level attained**	
Maximum of 9 school years	1
High school, vocational training	2
University degree (minimum Bachelor)	7
**Employment status**	
Full-time employed	6
Self-employed	1
Student	1
Sick leave	1
Retraining	1
**Highest BMI**	
35–40	1
40–45	3
45–50	4
50–60	1
>60	1
**Current BMI**	
20–25	1
25–30	5
30–35	1
35–40	3
**Weight reduction in kilogrammes**	
50–100 kg	6
30–50 kg	4
**Weight fluctuations**	
≤ ± 2 kg	5
≤ ± 5 kg	3
≥ ± 5 kg	2

### Recruitment

Eligible participants were identified through purposive sampling among individuals who had lost weight and kept it off over the long term after SO according to the defined inclusion criteria (Table 2; Given, 2008; Malterud et al., 2016).

**TABLE 2:** Study inclusion/exclusion criteria.

Inclusion	Previous BMI ≥40/35 with comorbidity	Current BMI ≤40/35 with comorbidity	Keeping off a minimum of 10% weight loss	≥5 years since current weight loss began
Exclusion	Bariatric surgery			

We received assistance from experienced family physicians and WL experts (typically dietitians who represented well-established companies offering guidance and support for lifestyle change and WL) to identify and recruit this selected group. They identified eligible participants and informed them about the study. If invited participants agreed to participate, the physician/dietitian/WL expert contacted the first author, and gave participants’ contact information. The first author sent information about the study and scheduled the interview. Identifying eligible participants was challenging; one might speculate that this subgroup of successful weight loss maintainers are largely healthy and self-directed as well as (perhaps to a lesser extent) supported by physicians and WL experts. Potential participants who had undergone BS or who had shorter duration of WL were excluded because *non-surgical* WL and long-term perspectives are essential specifications of the study context. SO is related to stigma, blame and feelings of shame, and we were aware of the sensitivity of these issues throughout the research process.

### Interviews

In planning and conducting the interviews, we built on two areas of researcher knowledge. The first area is relational, pertaining to which processes allow for establishment of rapport and participant safety within the relatively short period an interview lasts (Binder et al., 2012). The second area is topical, employing research knowledge to inform which topics should, as a minimum, be covered in the interview schedule (Brinkmann and Kvale, 2015). The objective of an exploratory in-depth interview is to balance the two areas, to achieve both structure to expand on existing knowledge, and the here-and-know safety for the participant to voice her own experiences in a way that is not limited by researcher pre-conception (Malterud, 2001b) The in-depth interviews began by repeating the purpose and context of the study to participants and collecting their demographic information. This choice is grounded in the relational area, as it aids in establishing the participant as one who knows and reduces the level of anxiety in the situation. Participants were asked open-ended questions about their WL experiences, relationships with their own bodies, WLM habits and practices, their social lives and how they define health and a good life. These are areas mandated by topical knowledge from relevant research literature. Based on the then established rapport, the interviewer encouraged participants to provide concrete and rich descriptions of their experiences, rather than beliefs and opinions. This interview strategy presupposes established safety, and rests on the methodological premise that first person experiences provide richer data for analyses, whereas opinions and beliefs largely are subject to social processes and discourse that may limit their ability to provide fresh information in an interview for exploratory purposes. Based on the above methodological approach to data collection, a thematic interview guide was used for preparation and as a tool for structuring the interviews (Supplementary Appendix). The interviews lasted 92 minutes on average (range 59–124 min) and yielded a comprehensive dataset of nuanced descriptions of long-term WLM. The data consisted of 11 in-depth interviews, because one of the participants was invited to a follow-up interview. The interviews were conducted between December 2015 and August 2016 by EN (6 interviews), CM (4 interviews), and MR (1 interview). All of us are senior researchers with experience in obesity research and in-depth interviewing following the described methodological principles (Malterud, 2001b; Brinkmann and Kvale, 2015). EN and MR are experienced physiotherapists, JA has clinical experience from nursing and CM is a specialist in clinical psychology. We had no previous relationships with the participants.

### Analyses

The transcripts were analysed using systematic text condensation (STC), a rigorous and inductive procedure for the thematic analysis of interview data across cases (Malterud, 2012). STC includes de-contextualising, coding, synthesising, and re-contextualising text, and it follows a stepwise approach. The current analysis concentrated on participants’ experiences with taking action and the strategies that they employed for long-term WL and WLM after SO. (1) Total impression: each member of the research team read the interview texts separately. Then the team met, discussed, and agreed on preliminary themes that stood out as meaningful as to the research question. The first-author developed further the naming of the preliminary themes by dwelling more in the texts, and by discussing with one of the team members (CM). The themes were: “Ownership,” “Drive,” “Point of no return,” “Continuity,” “Life spaces,” Self-reinforcing,” “Support,” and “The weight.”

**FIGURE 1:**
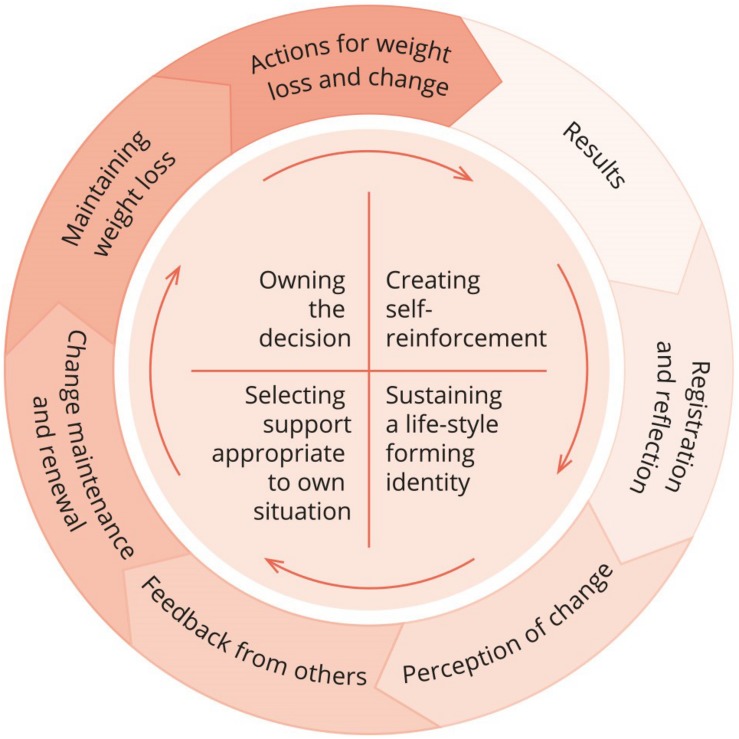
Experientially derived model of weight loss maintenance after severe obesity.

(2) Identification and sorting of meaning units: first-author coded the content of each interview according to the themes, which meant re-reading the texts, identifying and extracting “meaning units” that could enlighten each theme. The preliminary themes turned into code groups, where the content in each of them clearly had something in common. Underway, the code groups were adjusted as the first-author worked her way through the interview texts, reflecting on meaning units. What is this unit about? In which code group does it belong? Is there a need for another code group? Did the naming of code groups still catch the essence of the coded meaning units in them, or is the naming itself in need of change? This process was not linear, but went back and forth, and slipped into step 3, which is in accordance with STC recommendations. The need for reducing the number of code groups was an issue in this part of the process. For example, the first core theme was developed from “Ownership” and “The weight”. EN met with the co-authors once during this process.

(3) Condensation: the analysis moved forward through two integrated moves: further development of result categories (core themes) and writing condensed descriptions of core content in each of them. (4) Synthesising: first-author wrote presentations of the core themes, and included meaningful quotes from the interviews. The research team commented on this, and EN made some adjustments. The analysis ended with agreement on four final core themes (see section “Results”). The quality and richness of the material enabled further elaboration, particularly regarding the connections and interactions between intentionality and behaviour. A proposed conceptual model of WL and WLM after SO (Figure 1) was developed. This step represented the most abstract level of our analyses, and it was supported by self-determination theory (SDT) (Deci and Ryan, 2000, 2008b) and the work of Greaves et al. (2017) that focuses on psychological conflict/tension as a central concept of WLM and how it can be modified. STC enables a qualitative analysis to further develop concepts and theoretical models, depending on the materials, researchers, and status of knowledge (Malterud, 2017).

### Ethical Considerations

Talking to persons who have had SO about body weight, relationships, emotions and WLM can be a sensitive matter, demanding ethical reflection about the participants’ sense of mastery and well-being. The researchers in the group have longstanding clinical experience and the interviewers are experienced in interviewing people about SO and related issues. Participation was voluntarily with anonymity guaranteed. All participants provided written consent. The Norwegian Regional Committees for Medical Research approved this study (2012/1706/REK vest).

## Results

The results include four experiential core themes: (a) Owning the decision; (b) Creating self-reinforcement; (c) Sustaining a lifestyle-forming identity; and (d) Selecting support appropriate to one’s own situation. These themes constitute the level of intentionality. Here, intentionality points to the active, directed relation between the sociocultural world and our life, that “has two sides: there is the activity that interprets or grasps sensations and there is something of which I am conscious” (Slatman, 2014, p. 46). This means that intentionality is not merely what we think or intend to do; it is our embodied experience of the world trough perception, action, and interaction.

The core of the resulting model of WLM after SO describes the participants’ wholehearted commitment to conducting, renewing and sustaining radical life changes, and is both a foundation of and momentum for long-term WLM (Figure 1). The participants continued to take action for change, obtain results, record and reflect on their efforts and milestones, observed what worked and felt good, receive recognition from others and continue to change. The outer circle of the model concretises the behavioural level of manifesting the intentions, a system of meaningful practices, measures and perceptions that involves transformative experiences and personal values. Continuous transactions between the intentional- and behavioural level contributed to both persistence/stability and renewal/change among the participants’ experiences with WLM after SO.

### Owning the Decision

Successful WLM began when the participants realised the effect that SO has on their lives. All participants decided to lose weight and change their lives forever after recognising own life situation as impossible and unacceptable. They concluded that SO meant the risk of losing something valuable to them. For example, one woman experienced not being able to run after her children and feared having an accident.

*I have my life back. I can run after my children now; I can play with them, and I have so much energy. I could not do anything before because I used all my energy just to *be* [with emphasis] (Age 35–40, BMI 47.5–25.7)*.

The participants used their previous experiences with weight gain, WL, weight regain, diet, and exercise to plan their current life changes. WLM involved cognitive processes, a review of one’s own life, a plan and actions to change the situation. This intentional process both started and reflected the concrete actions that they undertook to successfully maintain WL. They wholeheartedly changed their dietary habits and levels of exercise for good. The actions taken reflected a commitment and engagement for lives and futures that were possible to enjoy and continue, without regaining weight.

*Although I have maintained my weight so far, it has fluctuated. I think I have to work with this [issue] for the rest of my life. I must keep creating the motivation. to keep my weight stable (Age 40–45, BMI 46.2–24.8)*.

Along with WL, establishing new eating habits, and PA routines, the participants changed their daily lives across several domains (e.g., by taking on new responsibilities). Some had switched jobs, whereas others had gained new competencies (e.g., a driver’s licence or a university degree). Some had established new relationships, one became a parent, and others built a new house. In this sense, WL, life change and flourishing seemed to converge.

*I have said to myself “NN, you have let your body become thick, and you just have to accept that it is going to fight you for a long time, but it is a battle worth fighting” because I feel so much better now than I did before (Age 35–40, BMI 40.6–27.0)*.

For the participants, WLM was both a meaningful challenge and an achievement worth fighting for.

### Creating Self-Reinforcement

Weight loss maintenance was both the cause and effect of effortful change. Self-reinforcement produced long-term WLM. Staying ready to reward, refine and renew life changes was helpful to avoid both distress and boredom.

*I had to take a hold of myself again and again and again. I did not exercise as I do now until these last years. I got rid of the weight, and then it became easier to work out. It felt better to move (Age 35–40, BMI 47.8–38.1)*.

Successfully losing weight after years of struggling with SO increased self-confidence and the ability to withstand change as well as to balance stability and renewal, ease and effort.

*I can see that it works out. One thing is being told [that it works], but you have to see it for yourself too… because I have succeeded. I have seen it; both physically and [stops] seen that my weight has dropped, and everything became easier…. It motivates me to continue. I am going down, for sure: 80 kilos is my main goal (Age 25–30, BMI 52.0–36.0)*.

Five years or more into WLM, the participants lived a regular and active daily life. To stay inspired and keep on track, most intentionally turned dieting and exercise into meaningful hobbies, and (for some) a new career path. They educated themselves, took courses, and joined networks of like-minded people with whom they shared their dedication, values and interests. Some became athletes and competed in cycling races, marathons, and triathlons.

*I walked home and found that there was quite a lot of strength in me. I felt [like I was] in better shape, as you do when [you] eat properly and decided to go for a walk on the same evening. And I continued, first every other day; then, I made a New Year’s resolution: I was going for a long walk every day for 100 days. And I ended up walking for 1,000 days (Age 30–35, BMI 66.8–39.1)*.

Self-monitoring was a means to stay motivated and in control of one’s changes. The participants kept track of a variety of indicators such as weight, physical performance, achievements, milestones, and before-and-after pictures. They used different accountability tools and encouragement strategies to continue the process, try new foods, new activities, and challenge themselves. One used a personal diary for exercise- and motivation; most used self-tracking devices, and some computed their statistics regarding WL, food consumption and exercise.

*I could not see it [weight and exercise outcomes] as others could, and I needed to see it. So, I used graphs. [I] plotted it into Excel, and then I could see how I did (Age 25–30, BMI 48.0–31.6)*.

Regarding becoming and staying healthy, the participants needed to build a structure. Some followed public dietary recommendations, whereas others “converted” to low carbohydrate/high fat/high protein diets. Having a diet that differed from conventional eating practices was complicated, but they experienced noticeable health benefits that reinforced their efforts. Participants experienced increased energy levels, the remission of severe migraine and abdominal symptoms, less musculoskeletal pain, satiation, and WL.

*I am confident that I know which diet suits me and that I can maintain my weight. I can still get sick, but I have hardly been ill after I switched diets. I do not catch a cold anymore, have no pain in my joints and such. My mind stays healthy. As soon as I let go a little, for example eating wheat, I felt it… feeling down and so on (Age 55–50, BMI 37.7–29.9)*.

The participants realised that they needed to work on their self-perceptions and advocated for WLM. A common strategy was to be prepared and identify situations that might trigger the impulse to overeat or settings where their diet was not possible to follow. Another strategy was social reinforcement; that is, spending time with people who take strong interest in healthy living and who supported their actions to maintain WL. Maintaining diet control and high exercise levels for years was rewarding but consumed most of the participants’ time and energy. Therefore, receiving support and positive feedback was crucial.

*Having a social life is something that I have gained, and it is a way to maintain my process of change. I need to be around people who are supportive and who know me, to do it and continue doing it (Age 30–35, BMI 66.8–39.1)*.

All participants described WL as a social breakthrough. After years of loneliness, some felt that they were building new relationships with friends and family, and others made the first moves toward romantic relationships. The participants described engaging with others and socialising in new ways as not only supportive of WL and change but also as an important dimension of personal change.

*Lonely years it was. [I] became isolated and withdrawn from [a social life], so I had to get back in, to learn something. Yes… learn talking to people again (Age 40–45, BMI 46.2–24.8)*.

### Sustaining a Lifestyle Forming Identity

Successful WLM entailed an active relationship with oneself and one’s own life trajectory. This process involves relating past experiences to the present situation and projecting into the future. Participants did not emphasise previous failures; rather, they used the insights that they had gained regarding losing weight and keeping it off successfully.

*I weigh myself once a month. The scale is creepy [quiet laugh] because I can become obsessed with it. I have been down that road before, stepping on the scale every day, so [short pause]…. It does not work out, and I fail (Age 40–45, BMI 46.2–24.8)*.

All participants had to stay focused and maintain healthy practices to avoid weight regain. They were highly attentive to everything concerning their diets, levels of daily activities and exercise, and how this made them feel and think in particular.

*Typically, visiting someone was a fight against having that piece of cake in the beginning. And knowing that I wanted the whole cake but not eating any. All the energy that you use to hold yourself back and not eat that cake… that is not an issue anymore. It is so much easier. I do not want the whole cake anymore (Age 35–40, BMI 43.0–26.2)*.

The participants linked their perceptions of feeling good about their bodies and selves with having a healthy lifestyle. Their lifestyle had gained new meanings, from a way to live to becoming who they were and wanted to be.

*It [exercise] means absolutely everything. I get in a terribly bad mood if I cannot get out.… To get out and be physically active is the alpha and omega of functioning, in fact. It is not about the weight *per se*, it is the psyche in general (Age 25–30, BMI 48.0–31.6)*.

Living completely changed lives involves both being proud of one’s self and being grateful. The participants cherished situations and experiences that confirmed their change and initiated feelings of success. For example, they experienced these feelings by remembering key moments from the process such as the experience of shopping for clothes when everything surprisingly fit and looked great on them. They recalled what this used to be like, how they had felt in fitting rooms before, and the contrast seemed to intensify the experience of change and not going back.

*When I lost so much weight, it was like a new world. I could go to the beach without being ashamed and use public changing rooms; it was not problematic at all. It was wonderful. Yes, it became a new world (Age 55–60, BMI 37.7–29.9)*.

### Selecting Support Appropriate to One’s Own Situation

The participants were the indisputable agents of successful WLM. They did not go entirely solo; rather, they actively selected the appropriate support at the right time guided by their needs. The most important support was that someone truly believed in them and their ability and capability to change their lives and lose weight over the long term. Support from managers and colleagues at work was essential and encouraged their efforts. For example, the participants were allowed to work from home occasionally or receive a temporarily more predictable work schedule.

*I talked to [my employers] at work, and they made some adjustments so that I could switch my shift or have a day off during the regular evenings that I went to the course. Then I felt safe that I could follow my course every week (Age 25–30, BMI 43.9–27.0)*.

Support from the family physician and other health practitioners throughout WL and WLM was crucial to some participants. Adequate support meant being there for them over the long term by listening, providing feedback and knowledge and being a collaborative partner during times of stagnation, illness or injury. Moreover, receiving the physician’s genuine interest and respect was described as inspirational.

*Without him [family physician], it would never have worked out because he explained my health to me… things that I had blocked out mentally quite effectively. When I was at my heaviest, it was hopeless. When I look back now, I made many excuses to myself all the time. Eventually, I wanted to do what it takes. He told me why it was so difficult for me to lose weight, about insulin resistance and all of that. Then I was able to start to research, slowly but surely, my own body and what felt right (Age 35–40, BMI 47.8–38.1)*.

Some participants had received a referral to visit a physiotherapist and/or a nurse working with health promotion in primary care and found what they needed there. However, most participants had received minimal support from their primary healthcare because practitioners could not provide the appropriate knowledge or support needed for long-term WLM after SO. They managed on their own by following a self-directed regimen or course, and some received online support from dieticians/physicians with specialised competencies and experience in diet and long-term lifestyle change. One exception was the participant who had been followed up extensively by primary healthcare during both her WL and WLM (due to pregnancy/regain).

## Discussion

The results are presented across four core themes that describe and concretise the experiences of self-directed WL and WLM following SO: (a) Owning the decision; (b) Creating self-reinforcement; (c) Sustaining a lifestyle forming identity; and (d) Selecting support appropriate to one’s own situation. Based on the results, we proposed a model of WLM after SO (Figure 1). Using the model, we suggest that flexible actions toward WLM on the behavioural level achieve their meaning and sustainability through their relationship with a core intentional level found across participants. One implication of this proposition is that this relationship between levels, and not the behaviours themselves, might be a meaningful conceptual focus when discussing WLM over the long term. Understanding an activity within the context of its intent may allow a stronger insight into its sustainability in long term WLM. For example, weighing food compliantly as mandatory part of a programme might have a very different meaning to an individual than weighing food to achieve nutritional control in building toward a long-term activity goal, such as running a marathon. We propose that understanding actions within their intentional context will provide meaningful insight into the phenomena they express.

Our analysis describes WL and WLM as integrated in experience, rather than as distinct phenomena. This hypothesis relates specifically to the group of participants who had previously been severely obese and, consequently, to the importance of WL and the losses and risks involved for participants in cases of weight regain. Some participants aimed to lose more weight, either because they had regained weight or because they had not lost enough in the first place. Thus, WLM was both cause and the effect of participants’ intentional approaches and simultaneously resulted from and enforced personal commitment.

The sense of managing WLM, being self-driven, tackling one’s weight and present weight challenges and using available support indicate *autonomous motivation*. SDT defines autonomous motivation as when people who identify with an activity’s value (e.g., exercise/diet compatible with WLM) are willing to integrate it into who they want to be, with a sense of genuine interest, choice and enjoyment (Deci and Ryan, 2000, 2008b). SDT suggests that people need to feel competent, autonomous and related to others, and social contexts that facilitate the fulfilment of these basic needs promote optimal motivation and tend to yield greater long-term persistence (Deci and Ryan, 2008a, b). The participants of the current study continuously adjusted their lives to accommodate WLM. Their experience demonstrates confidence in how to manage weight, a sense of control over important aspects of their lives and nurturing supportive relationships. Being alert and actively engaged in WLM and identifying with the activities and practices required to keep the weight off seem to not only result from but also cultivate autonomous motivation. These findings corroborate previous studies suggesting that feeling competent and autonomous enhances the opportunity to reach WL-related goals and long-term WLM (Teixeira et al., 2012, 2015; Gorin et al., 2014; Pedersen et al., 2018). The current study contributes to the empirical knowledge of autonomy and self-determination as relevant concepts in the context of long-term WLM.

Relating to others was essential to the experience of WLM across themes. Caring for one’s own children or establishing a romantic relationship or family made some decide to lose weight for good, and most participants developed a sense of belonging in new groups that were supportive of WL and WLM in terms of exercise and healthy living. Losing weight and keeping it off for 5 years brought challenges, and all participants needed encouragement and competent support from health practitioners; however, only some received the help that they needed. These findings are consistent with results from a study describing primary care patients with obesity in the United Kingdom (Evans et al., 2018). The main findings of that study were that WL was mostly self-directed, WL support was underutilised, people employ strategies that were not documented as effective and faced psychological and physical barriers. Consequently, people using self-directed approaches to WL and WLM (as opposed to interventions and programmes) should have access to professional support with competencies concerning WL and WLM.

In a systematic review of the qualitative research on WLM, Greaves et al. (2017) synthesised the results of 26 studies that included 710 participants who were successful or unsuccessful at maintaining WL, who were overweight, obese or severely obese, who had undergone bariatric surgery or who had used non-surgical approaches. Their resulting model suggested that the behaviour changes required for WLM generate psychological conflict/tension because of the need to set aside current habits and the discrepancy between the new behaviours and the fulfilment of psychological needs. Constant self-regulation, re-motivation and management of external influences likely reduce tension and increase the likelihood of successful WLM (Greaves et al., 2017). Addressing successful WLM in the context of SO and self-directed WL approaches, our study provided a thorough analysis of the strategies to manage WLM, and a parallel model was proposed that elaborated how tension can be managed, reduced and modified.

Because successful WL and WLM after SO interconnect and involve active, on-going and personal commitments through continuous transactions between intentional- and behavioural levels, the results and model inform the development of study designs and *non-surgical* interventions. The proposed model is based on people’s experiences of the possible implications of long-term WLM following SO as well as the demands on personal resources and support systems. Furthermore, this model contributes to the knowledge base that health professionals use when helping people with SO lose weight. For example, the conceptual model of intentions and behaviours could be tested for feasibility as a resource for people with SO seeking long-term WL. Responding to and discussing the model with peers and health professionals might strengthen and inspire people to find their own ways to WLM.

The current study explored a particular phenomenon within WLM (i.e., successful *non-surgical* WL and WLM among adults with previous SO) and concentrated on strategies designed to make and integrate the changes required for sustained WLM. Drawing on in-depth interviews and using a comprehensive and well-described strategy for data analysis, the results elaborate the current understanding of WLM. Our purposive sample of ten participants might be a limitation of this qualitative study. Identifying eligible participants for the current study was challenging, perhaps because we aimed for specific and long-term experiences of WLM. Success following self-directed WL is rare, and even more so among those with SO. However, we managed to obtain rich and varied data; moreover, we argue that the coherence among the specificity of the aims and study design, the comprehensive method of analysis and the application of theory enhances the study’s validity and rigour so that that a larger sample size is not required to provide strong findings (Whittemore et al., 2009; Malterud et al., 2016). The researcher’s subjectivity is addressed in qualitative research, acknowledging that different researchers might produce different but equally valid results; such diversity can contribute to a fuller understanding of complex phenomena (Malterud, 2001a; Finlay and Gough, 2003). The presence of our team of researchers involved in data production and analysis strengthened this study because it provided variation, multiple perspectives and informed discussions throughout the research process.

## Data Availability Statement

The datasets generated for this study will not be made publicly available due to ethical reasons. Data consists of interviews in Norwegian language transcribed verbatim, and the participants who consented have been ensured that raw data (their experiences/stories transcribed) will not be shared beyond the research team to protect their confidentiality. Requests to access the datasets should be directed to the corresponding author.

## Ethics Statement

The studies involving human participants were reviewed and approved by the Norwegian Regional Committees for Medical Research (2012/1706/REK vest). The patients/participants provided their written informed consent to participate in this study.

## Author Contributions

EN conceived the study, and led the data collection, analyses, and writing of the manuscript. EN, CM, and MR collected the data. All authors participated in the initial interpretation of findings (workshops) as well as read and approved the final manuscript.

## Conflict of Interest

The authors declare that the research was conducted in the absence of any commercial or financial relationships that could be construed as a potential conflict of interest.

## Abbreviations

PA: Physical activity
SO, Severe obesity: defined as having a body mass index ≥ 40 or ≥ 35 and obesity-related diseases
VLCD: Very low calorie diet
WL: Weight loss
WLM: Weight loss maintenance.
