# Supplementary material for: An Experientially Derived Model of Flexible and Intentional Actions for Weight Loss Maintenance After Severe Obesity
Source: Front Psychol. 2019 Nov 13;10:2503. doi: 10.3389/fpsyg.2019.02503 (PMC6863797; doi:10.3389/fpsyg.2019.02503)
Supplement: Supplementary file 1 [file Data_Sheet_1.PDF]

Thematic interview guide: Weight Loss and Weight Loss Maintenance after Severe Obesity

**Tema**	**Interview questions, potential follow-up questions**
**Opening, part of briefing**	*First, I would like to ask you a couple of questions concerning your background.*<br><br>Year of birth, marital/cohabiting status, family, housing, education, occupation, height, body weight (current, highest, lowest), magnitude of weight loss.
**Central topics**<br><br>Seeking concrete descriptions of lived experiences, situations and events.	«The weight loss journey» From severe obesity to successful weight loss maintenance.<br><br>***Can you tell about your weight trajectory throughout life?***<br><br>Weight gain, weight loss, weight regain, plateauing/stagnation, weight stability.<br><br>*Are there any other aspects of your life circumstances that are important?*<br><br>Specific events, experiences or situations that has evolved and related to the weight loss journey, or somehow have been meaningful.<br><br>Has your weight at any point been a threat to you, or your life situation?<br><br>Have you ever risked losing something that is valuable to you, related to your body weight?
**Own body (psyche and soma, relational)**	***How do you experience your own body in daily situations?***<br><br>The relational body, embodiment related to others, the body in the public space, the body undressed, intimacy, the body in movement.<br><br>*What has it meant for you that your body has become smaller?*<br><br>Esthetics, excess skin, movement, practices of everyday life, social encounters, participation/withdrawal, challenges.
**Meaningful habits**	***What changes have you made to your life to keep your body weight as it is now?***

	What has been important for you to carry out these changes over time?<br><br>*How do you relate to food and eating? How has this changed?*<br><br>*Can you tell about a day as it typically is, regarding food, meals, snacks and the like?*<br><br>*Can you tell about your movement and physical activity in an ordinary day/week? How is this at present? How was it before?*<br><br>*What does being in movement/ physical activity mean to you? How did it use to be?*<br><br>*How do you experience times allowing less focus on diet and physical activity? Can you tell about what you do in a such situation?*<br><br>*From where have you drawn support to manage long-term weight loss?*
**Participation/social encounters**	***How do these live changes affect your daily life and situations in which you are together with others?***<br><br>*How do these live changes affect your working life or situation related to education?*<br><br>*How has the changes affected you in your spare time?*<br><br>*How is your relationship to others?*<br><br>Partner, family, friends, colleagues or strangers.<br><br>*What has been the most important consequence of the life changes you have made and experienced, as you see it today?*
**Health (physical- and psychological)**	***What does "living the good life" mean for you? How do you experience your health at present, physical- and mental health?***<br><br>*What does the word «health» mean to you?*<br><br>*What other aspects, beyond life changes and weight loss, may have affected how you experience your health?*<br><br>*In case of persistent challenges related to health*

	and illness: Can you tell about how you live with it? Can you tell about an event or situation describing how you deal with your health problems on a good or a bad day? What do you think about the future?
**Summing up, closing, part of debriefing**	The interviewer sums up the main topics of the specific interview.<br><br>*Is there anything more you would like to tell or add about own weight loss journey that I have not asked about?*<br><br>*Is there anything more you would like to tell about own weight and body before we end this interview?*
